# Association of *Helicobacter pylori* Infection With Carotid Atherosclerosis in a Northern Chinese Population: A Cross-Sectional Study

**DOI:** 10.3389/fcvm.2021.795795

**Published:** 2022-01-31

**Authors:** Pu Zhang, Qian He, Daiyu Song, Yiying Wang, Xinyue Liu, Guoyong Ding, Weijia Xing

**Affiliations:** Department of Epidemiology, School of Public Health, Shandong First Medical University and Shandong Academy of Medical Sciences, Tai'an, China

**Keywords:** *Helicobacter pylori*, plaque, cross-sectional study, risk factor, carotid atherosclerosis

## Abstract

Numerous studies have shown that *Helicobacter pylori* (HP) infection may be involved in the development of carotid atherosclerosis (CAS), but this conclusion is still controversial. The aim of this study was to explore whether there is a positive association between HP infection and CAS occurrence. We collected data on demographic characteristics, lifestyle, and disease history of the participants by questionnaire. We obtained clinical anthropometric data and blood samples of the participants from clinical examinations and laboratory work. The ^13^C urea breath test (^13^C-UBT) was performed to assess the HP infection status, and carotid ultrasonography was used to diagnose the CAS and plaque types. Univariate analysis and multivariate logistic regression were used to identify the relationship between HP infection and CAS. A total of 1,424 participants were recruited for this study. A total of 740 HP-positive individuals and 684 HP-negative individuals were identified, and 345 participants were diagnosed with CAS. The prevalence of CAS was higher in the HP-positive group (26.4%) than in the HP-negative group (21.7%) (*P* < 0.05). A significantly higher prevalence of carotid intima-media thickening, carotid plaque, and carotid stenosis was identified in the HP-positive group than in the HP-negative group (*P* < 0.05). There was no significant difference in the detection rate of unstable plaques between the HP-positive and HP-negative groups (*P* > 0.05). In multivariate models adjusted for covariates, HP infection showed a positive association with CAS, independent of other risk factors (ORs range: 1.283–1.333, *P* < 0.05). HP infection independently accounted for approximately 5% of the CAS risk in the absence of other cardiovascular risk factors. A positive association between HP infection and CAS was demonstrated in this study. HP infection might be an independent risk factor for CAS. Although the effect of HP infection on CAS observed in our study was less than that of traditional risk factors, we believe that this is an indispensable advance in the etiological study of CAS. These results imply that the microbial population might play an essential role in CAS, which provides a new perspective for the primary prevention of CAS.

## Introduction

The increasing prevalence of carotid atherosclerosis (CAS) and cardio-cerebrovascular diseases (CVDs) in middle-aged adults and seniors imposes a heavy disease burden on society. CAS is characterized by endothelial injury and lipid deposition in carotid arteries, accompanied by thrombosis, fibrous tissue hyperplasia, and calcinosis, which can develop into atherosclerotic plaques (1). Plaque thickening can eventually lead to vascular occlusion and trigger ischemic cerebrovascular accidents (CVAs). Although past studies have explored the causes of atherogenesis, the results remain incomplete. Classic risk factors, such as age, sex, smoking, blood pressure, diabetes, high-density lipoprotein-cholesterol (HDL-cholesterol), and certain medications, account for 19.5% of the total plaque area burden (2). Identifying potential risk factors for CAS that are unexplained by traditional risk factors might improve the preventive strategies.

Over the past decade, there has been an increasing awareness of the potential link between infectious pathogens represented by *Helicobacter pylori* (HP) and atherosclerosis (3). HP is a microaerophilic Gram-negative bacterium colonizing the gastric epithelium. In China, the HP infection rate of the population in most areas exceeds 50% (4). HP can damage the gastric mucosal barrier and induce various gastrointestinal diseases, including chronic gastritis, peptic ulcers, and even gastric cancer (4). Although it colonizes the digestive tract, increasing evidence has shown that HP is associated with extragastric diseases such as CVD. However, some researchers have different perspectives on the existence of an association between HP and CAS (5). This is a controversial topic in epidemiological, pathological, and clinical studies (6).

CAS plays a role in the entire process from the subclinical phase to clinical CVD that is initially insidious until an ischemic accident occurs (7). Subclinical atherosclerosis is an early indicator of CVD, and identifying asymptomatic individuals is particularly important to reduce the disease burden (8). Previous studies tended to choose patients with clinical symptoms of atherosclerosis, and few studies focused on asymptomatic populations. Thus, we conducted a cross-sectional study to screen potential atherosclerotic patients and HP-infected populations, investigate the prevalence of CAS and HP infection in middle-aged individuals and seniors in northern China, and explore the association between HP infection and CAS.

## Materials and Methods

### Study Population

**Figure 1:**
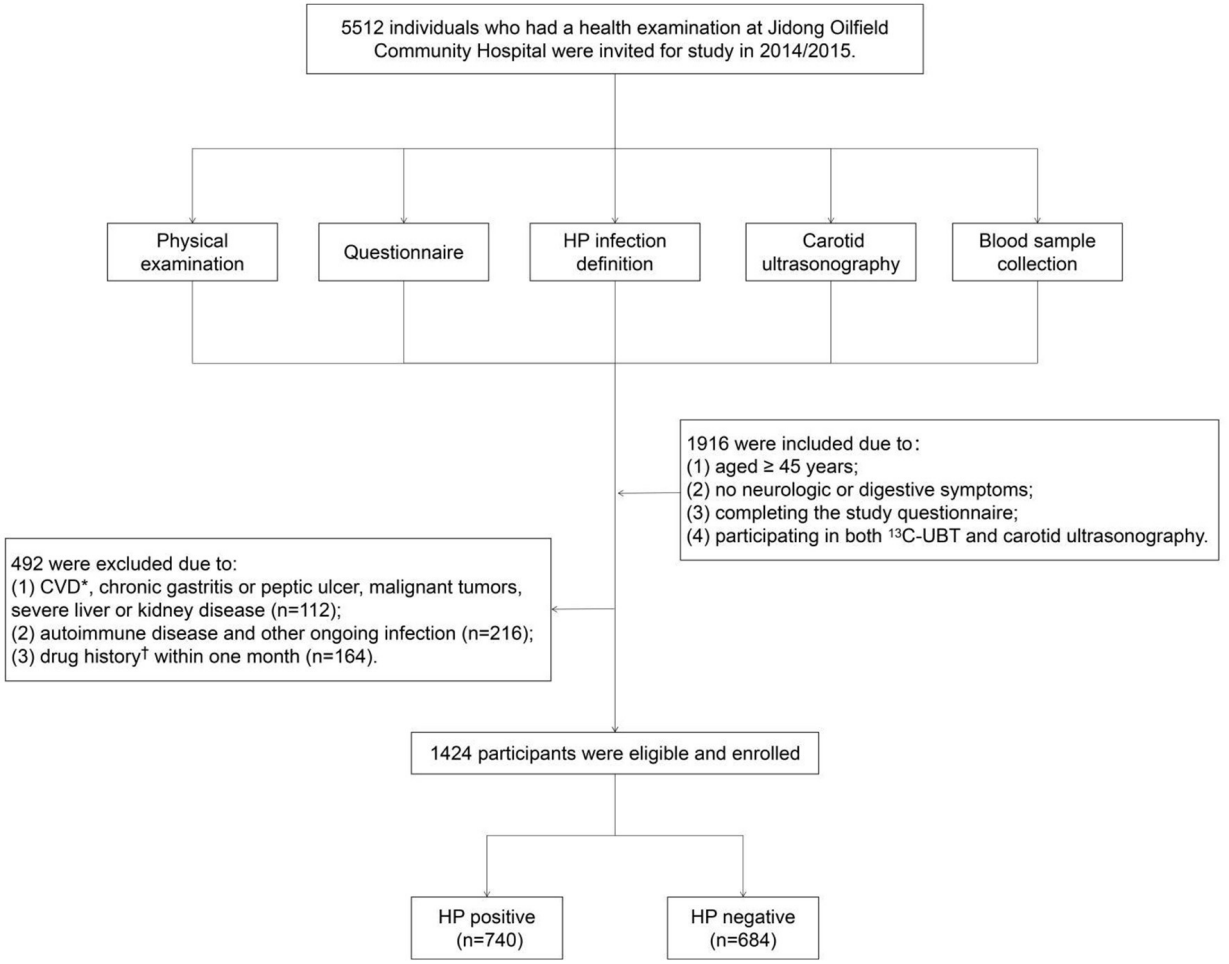
Flow chart of the study population enrollment. *Including coronary heart disease, myocardial infarction, atrial fibrillation, heart failure, and stroke. Drug history including aspirin, antibiotics, lipid-modulating drugs, and hormones. CVD, cardio-cerebrovascular diseases; HP, *Helicobacter pylori*.

A cross-sectional study was conducted in Jidong Oilfield Community Hospital (Caofeidian District, Tangshan City, Hebei Province, China) from Jan. 1, 2014 to Dec. 31, 2015. A total of 5,512 participants were recruited for the questionnaire and clinical anthropometrics. However, the participants were not obliged to undergo all of the examinations, such as a physical examination, blood sample collection, ^13^C urea breath test (^13^C-UBT), and carotid ultrasonography. Therefore, the inclusion criteria were defined as follows: (1) aged ≥ 45 years; (2) normal mental and intellectual status; (3) completing the study questionnaire; (4) participating in both ^13^C-UBT and carotid ultrasonography; and (5) no neurological symptoms or only nonspecific manifestations such as dizziness, headache, and syncope. In consideration of potential confounding factors, we excluded the following population: (1) patients with CVD, chronic gastritis or peptic ulcer, malignant tumors, severe liver or kidney disease (i.e., progressive liver cirrhosis, severe hepatitis, and chronic renal failure); (2) patients with autoimmune diseases or an ongoing infection; and (3) patients exposed to a drug history within 1 month. Finally, 1,424 participants were enrolled in our study (Figure 1).

### Case Definition

#### HP Infection

We performed ^13^C-UBT (HG-IRIS13C Infrared Spectrometer, Beijing Richen-Force Science & Technology Co. Ltd., Beijing, China) to determine the HP infection status of the participants. All participants fasted for more than 6 h before the test. Each subject orally consumed the drug containing ^13^C-urea. We collected the subject's baseline breath samples before dosing and postdose breath samples 30 min after dosing. After collection, two breath samples were tested simultaneously to assess the change in the ^13^C isotope abundance of expired carbon dioxide. HP positivity can be diagnosed by a delta-over baseline (DOB) value ≥ 4 (9).

#### Carotid Atherosclerosis

Carotid intima-media thickness (IMT) is defined by the distance between the aortic intima and the media adventitia (10). Participants underwent carotid Doppler ultrasound (EME Companion, Nicolet, Madison, WI, USA) to detect the lesions in the bilateral common carotid artery and internal carotid artery. The average IMT of three separate measurements was taken as the final result for each subject. According to the recommendations of radiologists, CAS was diagnosed by one of the following situations (11–14):

1. Carotid intima-media thickening: IMT ≥ 1.0 mm;
2. Carotid plaque: regional intimal thickening > 1.4 mm in height or double the adjacent IMT height;
3. Carotid stenosis: the degree of lumen stenosis ≥ 50%, with or without hemodynamic changes.

#### Atherosclerotic Plaque Type

According to the ultrasonic echoes reflected by the different components, the plaques can be divided into unstable plaques and stable plaques. The diagnostic criteria were as follows (15):

1. Stable plaque: hyperechoic plaque on ultrasonography, is characterized by a thick fibrous cap, macrocalcification, and extensive fibrous tissue, such as a fibrous flat plaque or calcified hard plaque;
2. Unstable plaque: hypoechoic plaque or inhomogeneous echoic plaque on ultrasonography, is characterized by a thin fibrous cap, large necrotic core, inflammatory response, plaque angiogenesis, intraplaque hemorrhage, and microcalcification, such as a lipid soft plaque or ulcerative mixed plaque.

#### Metabolic Syndrome

Metabolic syndrome refers to a combination of several interrelated CVD risk factors, including insulin resistance, obesity, atherogenic dyslipidemia and hypertension (16). It was diagnosed if three or more of the following conditions matched (17, 18):

1. Central obesity (waist circumference ≥ 90 cm in men and ≥ 85 cm in women) or BMI ≥ 25;
2. Systolic/diastolic blood pressure ≥ 140/90 mmHg or hypertension history;
3. Fasting blood glucose ≥ 6.1 mmol/L, 2 h plasma glucose ≥ 7.8 mmol/L, or a diabetes history;
4. Serum triglycerides ≥ 1.7 mmol/L, serum HDL-cholesterol <0.9 mmol/L in men and <1.0 mmol/L in women, or a dyslipidemia history.

### Questionnaire Investigation

A questionnaire was designed for the face-to-face interviews. The questionnaire collected information including demographics, lifestyle, and disease history. The demographic data included sex, age, educational level, marital status, and income level; lifestyle included smoking, alcohol consumption, physical activity, and salt intake levels; disease history included hypertension, hyperlipidemia, diabetes, CVD, malignant tumor, and autoimmune disease. The definitions of some characteristics were as follows: (1) physical activity was defined as regular moderate intensity exercise (i.e., the regular exercise with oxygen consumption reaching 60–70% maximal oxygen uptake) (19), and lack of physical activity was defined as moderate intensity physical activity <150 min per week; (3) smoking was defined as current smoking or quitting smoking for no more than 12 months; (4) alcohol consumption was defined as more than “one standard drink” (i.e., equivalent of 14 g pure alcohol) for each day; (5) excessive salt intake was defined as a total consummation of salts more than 6 g per day.

### Measurement of Blood Biochemical Indexes

The hematological tests were conducted on samples at the Central Laboratory of Kailuan General Hospital and Jidong Oil-Field Hospital, China. Blood samples of fasting participants were collected from the antecubital vein and stored in vacuum tubes containing ethylenediaminetetraacetic acid and coagulation tubes. The fasting blood glucose was measured using the hexokinase/glucose-6-phosphate dehydrogenase method. The cholesterol and triglyceride concentrations were determined by enzymatic methods (Mind Bioengineering Co., Ltd., Shanghai, China). The levels of apolipoprotein A, apolipoprotein B, HDL-cholesterol, and LDL-cholesterol were measured using an auto-analyzer (Hitachi, Tokyo, Japan) (20).

### Physical Examination

Physical examination included measurements of blood pressure, height, waist circumference, and weight, brachial-ankle pulse wave velocity (baPWV) examination, and ankle brachial index (ABI) examination. Three systolic blood pressure (SBP) and diastolic blood pressure (DBP) readings were collected to calculate the average as the final blood pressure. ABI examination and baPWV examination were used for assessment of lower extremities and coronary arterial state. All examinations were performed by clinicians.

### Statistical Methods

The Kolmogorov–Smirnov test was used to measure the normality of variables. Continuous variables are expressed as the mean ± standard deviation (mean ± SD) for normally distributed data, or the median and interquartile range for non-normally distributed data, and categorical variables are expressed as frequency and percentage (n, %). We analyzed the difference in the mean value or rate of each characteristic between the HP-positive and HP-negative groups by *t*-test, rank sum test, or χ^2^ test We used logistic regression analysis to examine the association between CAS and HP infection, and multivariate regression models were adjusted for covariates. All statistical tests were 2-sided, and differences with *P* < 0.05 were considered statistically significant. Statistical analysis software included SPSS 26 (Chicago, IL, USA), R 4.0 (Lucent Technologies, State of New Jersey, USA), and GraphPad Prism 8 (GraphPad Software Inc., La Jolla, CAS, USA).

## Results

### Baseline Characteristics of the Participants

We selected 1,424 participants aged 45–80 years, including 671 men (47.1%) and 753 women (52.9%). The average age of the participants was 56.6 years old (Table 1). Among them, 740 (51.97%) participants were HP positive, and 684 (48.03%) participants were HP negative. There were no statistically significant differences between the HP-positive and HP-negative groups in the comparison of baseline characteristics (*P* > 0.05).

**Table 1:** Comparison of baseline characteristics in participants with different statuses of HP infection.

**Characteristics**	**Total**	**HP positive**	**HP negative**	***P* value**
	***N* = 1424**	***N* = 740**	***N* = 684**	
Age, mean ± SD	56.6 ± 7.35	56.6 ± 7.48	56.5 ± 7.23	0.725
Male, *n* (%)	671 (47.1)	356 (46.9)	315 (51.0)	0.438
Educational levels, *n* (%)				0.113
Junior high school or below	132 (9.2)	75 (10.1)	57 (8.3)	
High school	901 (63.3)	478 (64.6)	423 (61.8)	
College or above	391 (27.5)	187 (25.3)	204 (29.8)	
Physical activity, i (%)				0.514
<150 min/week	491 (34.5)	261 (35.3)	230 (33.6)	
≥150 min/week	933 (65.5)	479 (64.7)	454 (66.4)	
Marital status, *n* (%)				0.624
Married	1,379 (96.8)	715 (96.6)	664 (97.1)	
Not married^*^	45 (3.2)	25 (3.4)	20 (2.9)	
Monthly income, *n* (%)				0.357
≤ 3,000 CNY^†^	817 (58.1)	432 (59.1)	385 (57.1)	
3,001-5,000 CNY	528 (37.6)	264 (36.1)	264 (39.2)	
>5,000 CNY	60 (4.3)	35 (4.8)	25 (3.7)	
Salt intake levels, *n* (%)				0.963
Low (<6 g)	317 (22.3)	164 (22.2)	153 (22.4)	
Medium (6–12 g)	736 (51.7)	385 (52.0)	351 (51.3)	
High (≥12 g)	371 (26.0)	191 (25.8)	180 (26.3)	
Smoking, *n* (%)	389 (27.3)	216 (29.2)	173 (25.3)	0.099
Alcohol consumption, *n* (%)	399 (28.0)	220 (29.7)	179 (26.2)	0.135

* *Including separated, single, widowed, and divorced*.

† *1 CNY approximately equals to 0.1572 dollar*.

*HP, Helicobacter pylori*.

### Disease and Clinical Indicators of the Participants

A total of 345 (24.23%) were diagnosed as CAS patients. A total of 196 (26.4%) and 149 (21.7%) CAS patients were diagnosed in the HP-positive and HP-negative groups, respectively (Table 2). The prevalence of CAS was higher in the HP-positive group than in the HP-negative group, and the differences were statistically significant (26.4 vs. 21.7%, *P* < 0.05). There were no statistically significant differences between the two groups for any other characteristics (*P* > 0.05).

**Table 2:** Comparison of disease and clinical indicators in participants with different statuses of HP infection.

**Characteristics**	**Total**	**HP positive**	**HP negative**	***P* value**
	***N* = 1,424**	***N* = 740**	***N* = 684**	
Metabolic syndrome, *n* (%)	589 (41.4)	313 (42.3)	276 (40.4)	0.456
Carotid atherosclerosis, *n* (%)	345 (24.2)	196 (26.4)	149 (21.7)	0.039
Diabetes mellitus, *n* (%)	178 (12.5)	97 (13.1)	81 (11.8)	0.470
BMI (kg/m^2^), mean ± SD	25.0 ± 3.24	25.2 ± 3.23	24.9 ± 3.25	0.096
FBG (mmol/L), mean ± SD	6.39 ± 1.64	6.42 ± 1.63	6.36 ± 1.66	0.459
baPWV^*^ (cm/s)	1,535 ± 294.49	1,531 ± 291.58	1,541 ± 298.11	0.513
ABI	1.15 ± 0.08	1.16 ± 0.09	1.15 ± 0.08	0.261
Blood pressure, mean ± SD				
SBP (mmHg)	133 ± 46.99	134 ± 62.61	131 ± 18.56	0.277
DBP (mmHg)	83 ± 12.19	83 ± 12.41	82 ± 11.96	0.722
Lipid parameters^*^, M (IQR)				
ApoA (g/L)	1.34 (0.16)	1.33 (0.15)	1.34 (0.17)	0.636
ApoB (g/L)	1.01 (0.18)	1.01 (0.18)	1.00 (0.17)	0.377
LDL-cholesterol (mmol/L)	3.50 (0.79)	3.51 (0.80)	3.48 (0.79)	0.484
HDL-cholesterol (mmol/L)	1.28 (0.28)	1.27 (0.27)	1.29 (0.29)	0.084
TC (mmol/L)	5.25 (0.96)	5.26 (0.99)	5.23 (0.93)	0.564
TG (mmol/L)	1.96 (1.40)	2.02 (1.43)	1.88 (1.37)	0.062

*ABI, ankle brachial index; ApoA, apolipoprotein A; ApoB, apolipoprotein B; baPWV, brachial-ankle pulse wave velocity; BMI, body mass index; FBG, fasting blood glucose; HDL-cholesterol, high-density lipoprotein-cholesterol; IQR, interquartile range; LDL-cholesterol, low-density lipoprotein-cholesterol; M, median; SD, standard deviation; TC, total cholesterol; TG, triglyceride*.

* *17/1,424 subjects refused to collect their blood sample, the missing data accounted for 1.2%*.

### Independent Variable Screening for Multivariate Analysis

**Figure 2:**
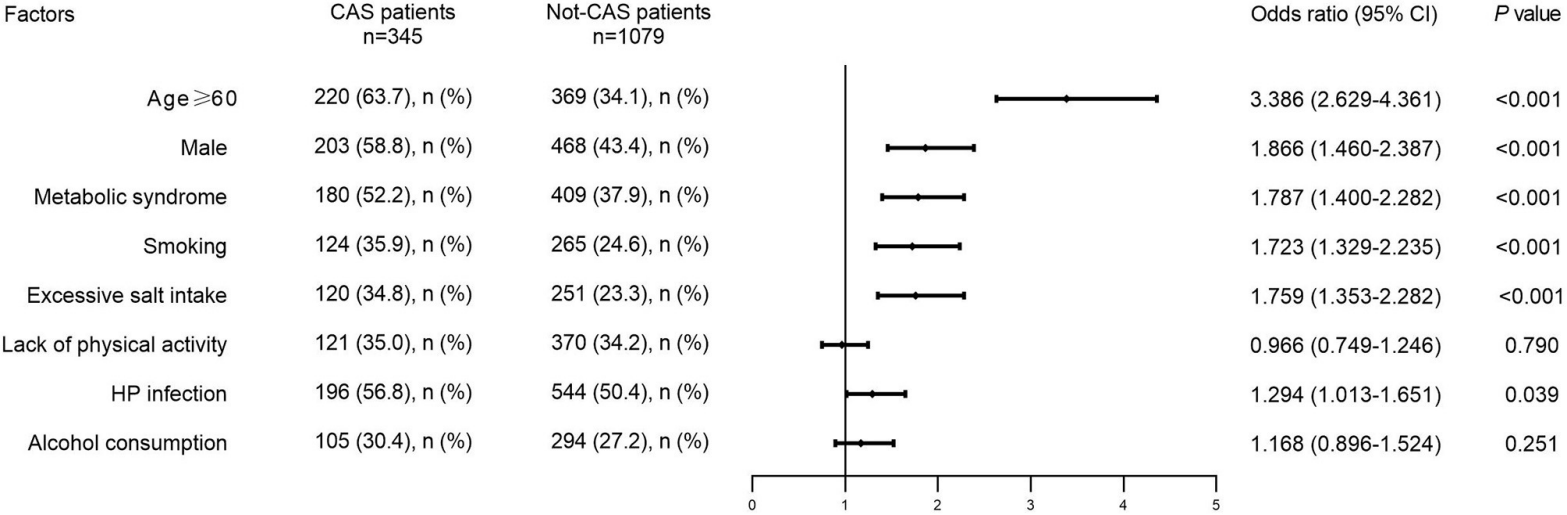
Distribution of potential risk factors in CAS patients and not-CAS patients. CAS, carotid atherosclerosis; HP, *Helicobacter pylori*.

Baseline data analysis revealed an association between HP infection and CAS (OR = 1.294, 95% CI: 1.013–1.651), but the effects of other factors could not be ignored. Increased age, sex differences, excessive salt intake, smoking, metabolic syndrome, alcohol consumption, and a lack of physical activity were relevant factors for CAS. After univariate analysis, variables including age ≥ 60 (OR = 3.386, 95% CI: 2.629–4.361), males (OR = 1.866, 95% CI: 1.460–2.387), excessive salt intake (OR = 1.759, 95% CI: 1.353–2.282), smoking (OR = 1.723, 95% CI: 1.329–2.235), and metabolic syndrome (OR = 1.787, 95% CI: 1.400–2.282) were selected for inclusion in multivariate models (*P* < 0.05), while alcohol consumption (OR = 1.168, 95% CI: 0.896–1.524) and lack of physical activity (OR = 0.966, 95% CI: 0.749–1.246) were excluded (*P* > 0.05) (Figure 2).

### Association Between HP Infection and CAS

**Figure 3:**
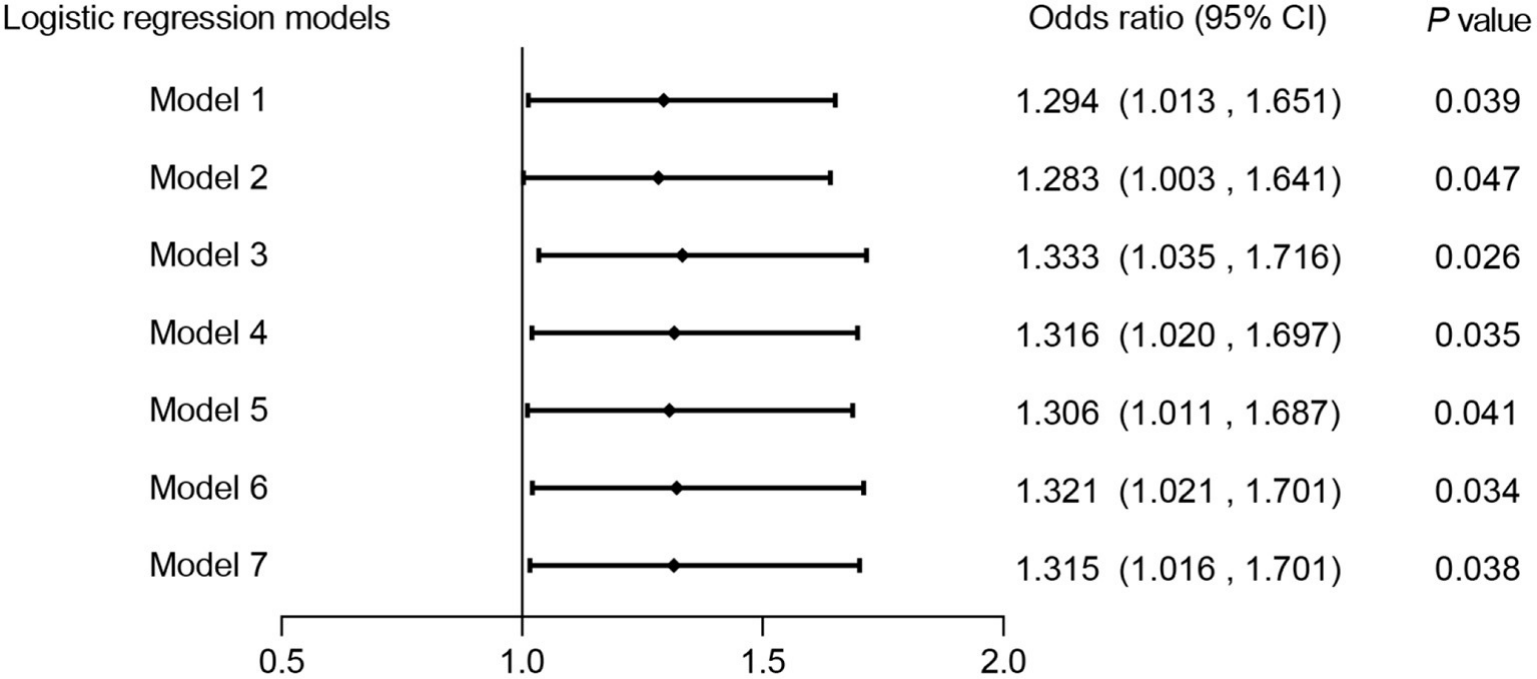
Association between HP infection and CAS in different adjusted multivariate logistic regression models. Model 1: unadjusted. Model 2: adjusted for sex. Model 3: adjusted for age. Model 4: adjusted for age and sex. Model 5: adjusted for age, sex and metabolic syndrome. Model 6: adjusted for age, sex, metabolic syndrome and excessive salt intake. Model 7: adjusted for age, sex, metabolic syndrome, excessive salt intake and smoking. CAS, carotid atherosclerosis; HP, *Helicobacter pylori*.

Seven adjusted multivariate logistic regression models were used to identify the potential association between Hp infection and CAS prevalence. As Figure 3 shows, HP infection had a positive relationship with CAS in all covariate-adjusted multivariate models, suggesting that HP infection might be an independent risk factor for CAS (ORs range: 1.283–1.333, *P* < 0.05).

**Figure 4:**
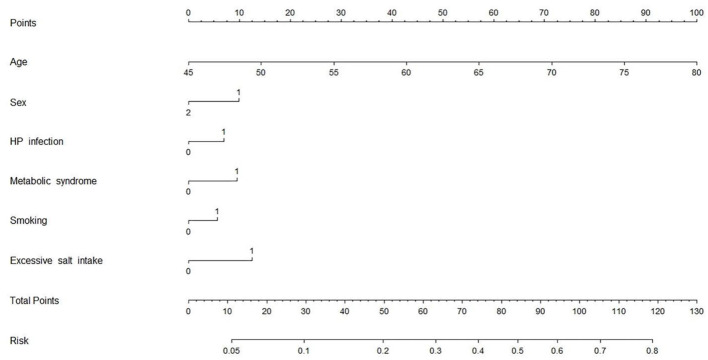
CAS risk prediction model. Variable definition. Sex: 1 = male, 2 = female; HP infection: 0 = negative, 1 = positive; Metabolic syndrome: 0 = no, 1 = yes; Excessive salt intake: 0 = no, 1 = yes; Smoking: 0 = no, 1 = yes. CAS, carotid atherosclerosis; HP, *Helicobacter pylori*.

Combining HP infection and classical risk factors, we designed a multivariate risk model in the form of a nomogram to predict the risk of CAS (Figure 4). To use the nomogram: (1) find the position of each variable on the corresponding axis; (2) draw a vertical line to the *Points* axis to get the score of each variable; (3) add up the scores of each variable to get a total score and find it on the *Total Points* axis; (4) draw a vertical line from the *Total Points* axis to the *Risk* axis to determine the CAS risk (21). It was found that HP infection can explain approximately 5% of the CAS risk when the other factors are negative.

### Association Between HP Infection and Three Types of CAS

**Figure 5:**
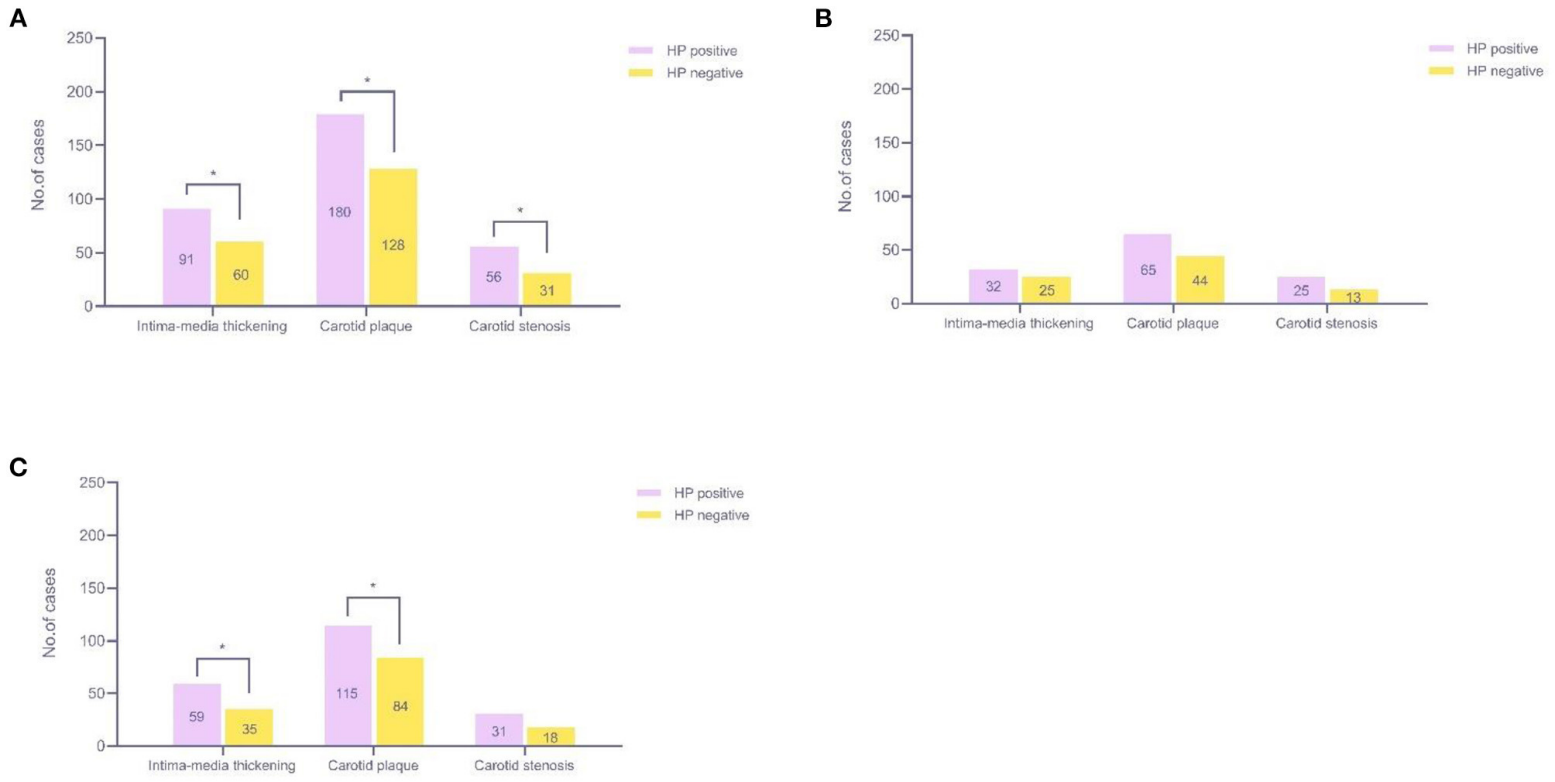
Relationship among the three types of CAS and HP infection. **(A)** All participants (45–81 years old, *n* = 1,424); **(B)** Middle-aged participants (45–59 years old, *n* = 589); **(C)** Seniors (over 60 years old, *n* = 835). *P < 0.05. CAS, carotid atherosclerosis; HP, *Helicobacter pylori*.

We detected 151 patients with carotid intima-media thickening, 308 patients with carotid plaques, and 87 patients with carotid stenosis. The number of plaque patients was the highest. Among all participants, we found a positive association between HP infection and each type of CAS; the HP-positive group had a higher prevalence of carotid intima-media thickening, carotid plaque, and carotid stenosis than the HP-negative group, and the difference was statistically significant (12.3 *vs*. 8.8%, 7.6 *vs*. 4.5%, 24.3 *vs*. 18.7%, *P* < 0.05) (Figure 5A). Since age was the most important confounding factor, we performed further analysis by age stratification. As Figure 5B shows, no statistically significant differences were observed in the middle-aged group (*P* > 0.05). In seniors, the HP-positive group had a higher prevalence of carotid intima-media thickening and carotid plaque than the HP-negative group, and the difference was statistically significant (19.5 *vs*. 12.2%, 38.0 *vs*. 29.4%, *P* < 0.05) (Figure 5C).

### Association Between HP Infection and Atherosclerotic Plaque Types

**Figure 6:**
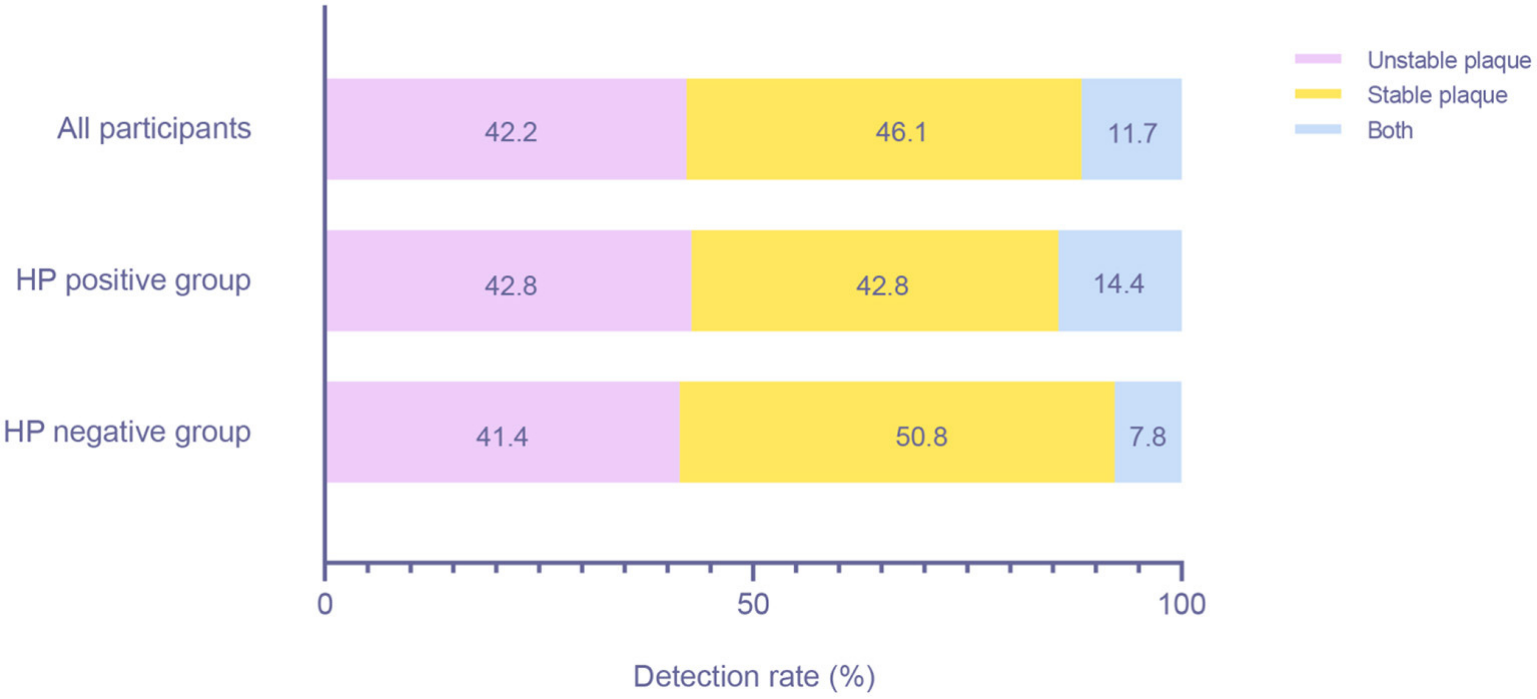
Detection rates of patients with different plaque types between the HP-positive and HP-negative groups. HP, *Helicobacter pylori*.

We identified a total of 308 patients with plaques. A total of 130 (42.2%) patients had unstable plaques, 142 (46.1%) patients had stable plaques, and 36 (11.7%) patients had both types of plaques (Figure 6). There were 77 (42.8%) unstable plaque patients, 77 (42.8%) stable plaque patients and 26 (14.4%) mixed patients in the HP-positive group and 53 (41.4%) unstable plaque patients, 65 (50.8%) stable plaque patients and 10 (7.8%) mixed patients in the HP-negative group. However, we found no significant difference in the detection rate of different plaque types between the HP-positive and HP-negative groups (*P* > 0.05).

## Discussion

Atherosclerosis, one of the pathological bases of CVD, reveals the early stage of CVD. The etiology of atherosclerosis is complex. In addition to the identified risk factors, studies have proposed that HP infection may be a risk factor for CAS, but some researchers still hold the opposite opinion (5, 22, 23). We conducted a cross-sectional study of 1,424 subjects in Jidong from 2014 to 2015 to explore the association between HP and CAS. According to the different manifestations on ultrasound examination, we defined CAS as intimal thickening, plaque or stenosis. In this study, the HP-positive group had a higher prevalence of CAS than the HP-negative group, and this result was also found in various CAS types. We found that a positive relationship between HP infection and CAS existed independently of the classical risk factors, suggesting that HP infection might be one of the independent risk factors for CAS.

In China, CVA induced by CAS, such as ischemic stroke, has always been a primary cause of mortality for middle-aged adults and seniors, imposing a heavy disease burden on the country (24, 25). A meta-analysis showed that 27.22% of Chinese people aged 30~79 years had CAS (14), similar to the 24.23% reported in our study. Although many risk factors for atherosclerosis, such as sex differences, aging, smoking, obesity, blood pressure, blood cholesterol, and diabetes, have been identified (7, 14), these cannot fully explain all cases. Increasing evidence indicates that microbial infection might be a novel risk factor for atherosclerotic plaque formation or rupture (26). Our results support this conclusion. We found that HP infection can explain approximately 5% of CAS risk independently of traditional factors, which means that the role of microbial infection cannot be underestimated. The essence of atherosclerosis is an inflammatory lesion in arterial blood vessels, and inflammation is involved in all stages of initiation and progression of atherosclerotic plaques (27, 28). As a common pathogen causing chronic infection, HP can not only cause local lesions in gastric mucosa tissue but also persistently stimulate the immune response of the human body, producing a large number of inflammatory cytokines, which are also atherosclerosis-evoking cytokines (29, 30). As early as 2001, Ameriso et al. found that HP existed in carotid atherosclerotic plaques and was related to the features of the inflammatory cell response (31). Oshima et al. found that C-reactive protein (CRP) and inflammatory adhesion molecules were elevated in patients with HP infection, suggesting a possible link between HP infection and endothelial dysfunction (32). In addition, chronic HP infection increased the levels of cytokines such as tumor necrosis factor-α (TNF-α), interleukin (IL), γ-interferon (γ-IFN), coagulation factor-fibrinogen, and thrombin (33, 34), which are closely related to the formation of atherosclerotic plaques (28). Our study used different multivariate models to control for other factors and found an independent role of HP infection in CAS occurrence, which may lay a foundation for further mechanistic studies.

Carotid intima-media thickening, carotid plaque, and carotid stenosis are early markers of subclinical atherosclerosis and predictors of mortality and cardiovascular events (35). In this study, HP-infected individuals had higher levels of carotid intima-media thickness than HP-negative individuals, which is consistent with the findings of Başyigit et al. (36). Compared with HP-negative individuals, the prevalence of various CASs in HP-infected individuals was higher. However, after grouping by age, these associations are reflected only in seniors. Generally, the prevalence of increased carotid intima-media thickness, carotid plaque, and carotid stenosis all increased with increasing age (37). We speculate that because seniors have a higher CAS prevalence and a longer period of HP infection than middle-aged individuals, the impact of HP infection on the occurrence of CAS was more significant.

Recently, researchers have focused more on the instability of plaques, not just the existence of plaques. An unstable plaque containing lipid or hemorrhagic components was an independent risk factor for ischemic CVA (38–40). Gabrielli, Wu, Pietroiusti et al. demonstrated that infection with HP, especially cytotoxicity-associated gene-A (CagA)-positive HP strains, was associated with carotid plaque instability (41–44). However, our results did not support this conclusion. HP virulence factors vary depending on the geographical distribution, and not all HPs can produce these virulence proteins (45). Although the dominant HP isolates are CagA gene-positive in East Asia (45, 46), some infected individuals in our study may carry CagA gene-negative strains, and whether the negative strains affect the stability of plaques is still unclear, which may cause inaccurate results. In addition, we speculated that the bacterial content in the plaque potentially affects plaque stability, and when the bacteria are present in small amounts, they will not affect the stability of plaques.

Our study showed that the HP infection rate in middle-aged adults and seniors in northern China was 51.97%, close to the 53.0% reported by Zhang et al. (45). With improvements in sanitation, the HP infection rate has been showing a downward trend ADDIN EN.CITE (47), but the situation is still not optimistic. Although the reported HP infection rates vary depending on the geographical regions, population, age, and sample size, most studies in China have reported rates above 50% (45, 48). HP infection is usually latent, with only approximately 10% of infected individuals presenting with clinical symptoms (49, 50), which means that most patients may be unaware of their condition, posing a huge potential health hazard to the population. Furthermore, HP infection is difficult to eradicate and easily recurs (51–53). The problem is particularly difficult in China due to antibiotic resistance caused by excessive antibiotic consumption, genetic polymorphisms in drug metabolism enzymes, lack of patient compliance, and bacterial factors (45, 54). Therefore, from the perspective of public health, effective anti-HP treatment is crucial for the prevention and treatment of both gastric diseases and extragastric diseases.

### Highlights

Previous studies focused on symptomatic CAS patients and HP-infected people with gastrointestinal symptoms but ignored a large proportion of the asymptomatic population. In this study, we chose asymptomatic individuals as subjects and found they had a high prevalence of both HP infection and CAS, suggesting that more attention should be paid to screening asymptomatic individuals to control the deterioration in either condition. In addition, we quantitatively showed the proportion of HP infection in CAS risk through a nomogram, which has not been reported in previous studies. This can help readers understand the relationship between the two more intuitively.

### Limitations

The participants were from a small-scale district, which may be insufficient to represent the general population. Additionally, some participants did not complete all of the tests, which caused incomplete data and adversely affected the data analysis. Because of the cross-sectional design and insufficient data, we failed to obtain direct evidence to confirm the mechanism of HP infection in the occurrence and progression of atherosclerosis.

## Conclusion

The findings of this study suggest that HP infection has an independent positive association with CAS in middle-aged adults and seniors. Although a single HP infection plays a relatively minor role in CAS occurrence, the cumulative infectious burden, such as long-term or repeated infection, may significantly increase the risk of disease.

## Data Availability Statement

The data analyzed in this study is subject to the following licenses/restrictions: The dataset used in this study is privately owned. Requests to access these datasets should be directed to Weijia Xing, wjxing@sdfmu.edu.cn.

## Ethics Statement

The studies involving human participants were reviewed and approved by the Ethics Committee of the Staff Hospital of Jidong Oil Field of China National Petroleum Corporation (CNPC) (Tangshan, China). The patients/participants provided their written informed consent to participate in this study.

## Author Contributions

WX, GD, QH, and PZ: study design. DS, PZ, YW, and XL: data collection, analysis, and interpretation. PZ: manuscript writing. PZ, QH, and WX: statistical analysis and administrative, technical or material support, and supervision. WX: critical revision of the manuscript for intellectual content. All authors approved the final version of the paper.

## Funding

This study was supported by the National Natural Science Foundation of China, (No. 81903401), the Young Taishan Scholars Program of Shandong Province of China, (No. tsqn20161046), the Shandong Province Higher Educational Young and Innovation Technology Supporting Program (2019KJL004), and the Academic Promotion Programme of Shandong First Medical University, (No. 2019RC010).

## Conflict of Interest

The authors declare that the research was conducted in the absence of any commercial or financial relationships that could be construed as a potential conflict of interest.

## Publisher's Note

All claims expressed in this article are solely those of the authors and do not necessarily represent those of their affiliated organizations, or those of the publisher, the editors and the reviewers. Any product that may be evaluated in this article, or claim that may be made by its manufacturer, is not guaranteed or endorsed by the publisher.
